# An Unusual Psychiatric Presentation of the 3q29 Microduplication Syndrome

**DOI:** 10.7759/cureus.7203

**Published:** 2020-03-08

**Authors:** Filipa Reis, Cristina Pereira, Maria Laureano, Teresa Cartaxo

**Affiliations:** 1 Child and Adolescent Psychiatry, Centro Hospitalar e Universitário de Coimbra, Coimbra, PRT; 2 Neuropediatrics, Centro Hospitalar e Universitário de Coimbra, Coimbra, PRT

**Keywords:** child and adolescent psychiatry, 3q29 microduplication syndrome, intellectual disability, emotional dysregulation, differential diagnosis

## Abstract

The 3q29 microduplication syndrome is usually associated with an intellectual disability or global developmental delay and mild dysmorphisms. Other comorbid presentations reported in the literature include psychiatric disorders such as behavioral disorders, attention-deficit/hyperactivity disorders, elimination disorders, and autism spectrum disorders. The current case is of an adolescent girl with the 3q29 microduplication syndrome who had a diverse psychiatric presentation. The patient was a 14-year-old girl in institutional care, with a moderate intellectual developmental disorder, major behavioral problems, with auto- and hetero-aggressions and a suspicious trait, who presented with frequent episodes of emotional dysregulation, disorganized speech with derailment, incoherence, perseveration and grossly disorganized behavior. Auditory hallucinations were suspected sometimes but were difficult to evaluate. In our assessment, we were not able to determine a diagnosis because the symptoms do not seem to be defined by any classification. Major pharmacological and non-pharmacological interventions were needed to manage this case.

## Introduction

The 3q29 microduplication syndrome (Online Mendelian Inheritance in Man 611936) is usually associated with an intellectual disability or global developmental delay, language delay, mild facial dysmorphisms, congenital heart diseases, skeletal malformations, eye anomalies, and obesity [1-9]. The genes that contribute to the clinical features of this syndrome are not clear, but the range of the copy number variation goes from 1.6 to 2.3 Mb, including the transferrin receptor (TFRC) and 3-hydroxybutyrate dehydrogenase 1 (BDH1) genes [9].

To our knowledge, there are 35 cases of 3q29 microduplication syndrome reported in the literature [1-11].

Although the phenotype of this syndrome is variable, mild-to-moderate intellectual disability appears to be the most prominent feature [1]. Nevertheless, other neurodevelopmental and psychiatric disorders have been reported, including autism spectrum disorder, attention-deficit/hyperactivity disorder, and behavior disorders [1,5].

Here, we discuss an unreported psychiatric presentation of the 3q29 microduplication syndrome.

## Case presentation

The patient was a 14-year-old girl, with no neonatal information documented, placed in institutional care at the age of nine because of parental neglect. Two of her three brothers were also in care but in another facility, and the adult brother was incarcerated in prison. The parents are non-consanguineous, and the father has a substance use disorder. One of the brothers was described as having macrocephaly, hirsutism, and cognitive impairment. There was no other relevant medical family history.

There was no description of her developmental history before being taken into care, but at the time, she had already exhibited behavioral problems, with oppositional conduct to adults, hetero-aggression to peers, learning disabilities with school retention, nystagmus, simple motor tics, and tooth decay. These concerns motivated the referral to child and adolescent psychiatric, pediatric, oral medicine, and ophthalmology consultations at the age of nine.

The ophthalmologic evaluation determined that her symmetric horizontal and torsional bilateral nystagmus was congenital, with no impairment in her visual acuity. The pediatric examination excluded major organic disorders and referred her to a neurologic consultation because of the simple motor tics.

In the neurologic examination, the girl presented normal length (10th to 25th centile), weight (50th centile), and cephalic perimeter (50th centile), a long face, a short nose with a bulbous tip, thick eyelashes, upslanting palpebral fissures, and a hemangioma-like lesion on the posterior region of the right thigh. The major findings were persistent motor tic disorder (frequent shoulder elevation, cephalic rotation, and blinking, but no vocal tics), the already described nystagmus, a general clumsiness, and dysdiadokokinesis.

**Figure 1:**
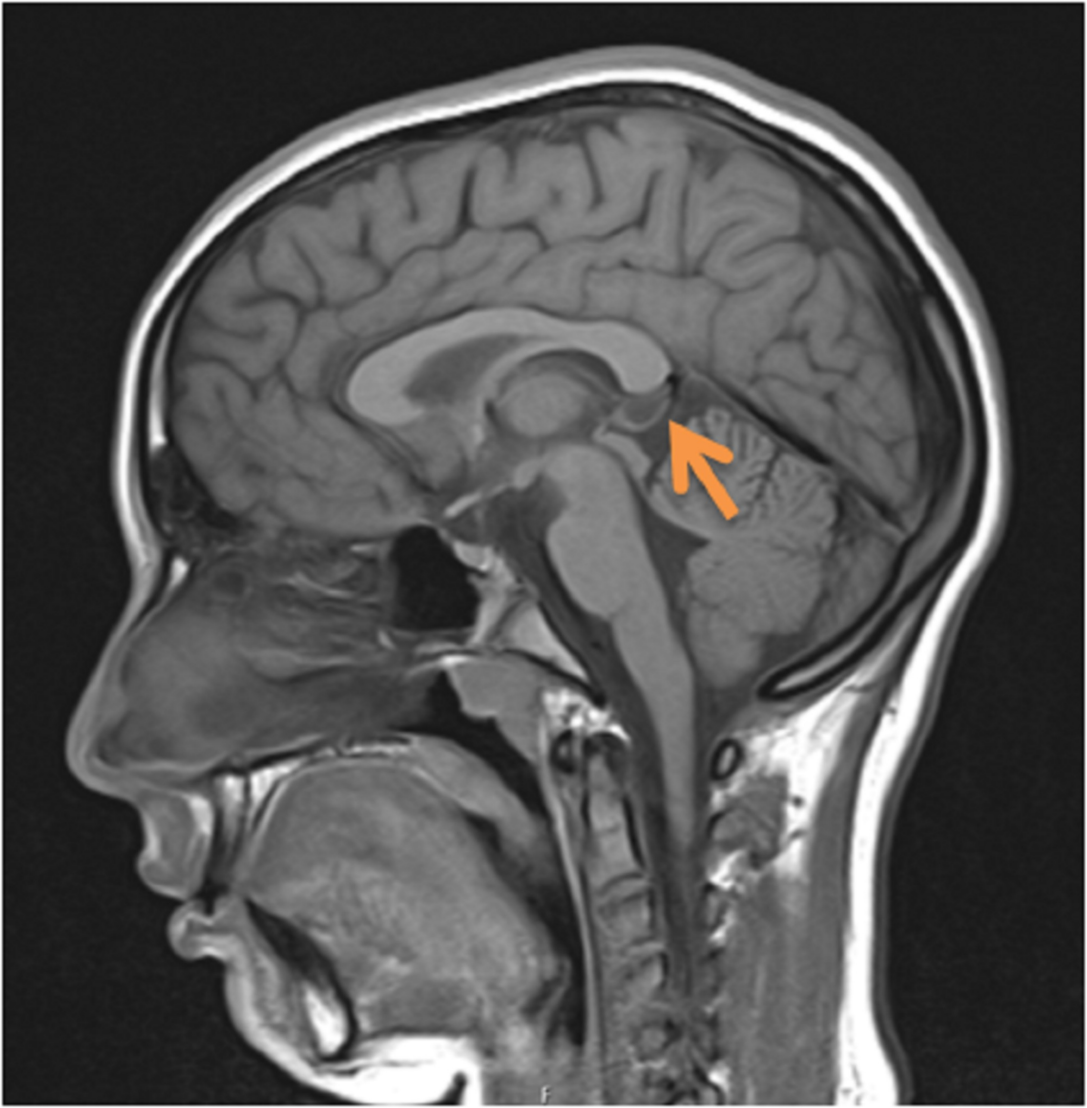
MRI brain sagittal section showing a small cyst of the pineal gland and no other relevant structural abnormalities.

Brain magnetic resonance imaging (MRI) was performed to exclude cerebellar disorders. It revealed a small cyst in the pineal gland (9.5 mm), and there were no other alterations. A second brain magnetic resonance imaging, one year later, was similar to the first one, and there were no signs of progressive cerebellar atrophy (Figure 1). Full blood count, biochemical profiles, metabolic, autoimmune and thyroid tests, and the copper metabolism study were also normal, excluding treatable causes of movement disorders. Finally, a genetic evaluation was requested, and the array-comparative genomic hybridization (array-CGH) detected a 3q29 microduplication. The brother with dysmorphisms was also tested and had the same genetic abnormality. Only the mother consented to the evaluation, which had no alterations in this chromosome. Two vigil and sleep video electroencephalographies were also normal.

The initial psychiatric evaluation at the age of 10 suspected an intellectual disability, with repercussions in her academic achievements (school retention) and adaptive functioning skills (poor social skills, speech delay, and lack of autonomy). The psychological evaluation with the Wechsler Intelligence Scale for Children (WISC-III scale) showed a total intelligence quotient (IQ) of 48 [12]. She required academic adaptations, speech therapy, psychomotor therapy, and extra help and supervision in her daily life activities, confirming moderate intellectual developmental disorder.

Initially, with behavioral techniques, emotional support, and parental, supervised visits, the girl reduced her aggressive behavior toward others and was able to achieve academic milestones (in reading and writing). However, her caretakers frequently mentioned persecutory ideation in the girl (a frequent belief that she was being spied on or talked about).

Afterward, progressive parental negligence, including missed visitations and calls, with a consequent impact on the girl, worsened her behavior, with hetero-aggression and disrupted conduct.

At the age of 11, she developed a major depressive disorder after a tribunal-imposed contact with her parents, leading to major behavioral dysregulation, with irritable mood, psychomotor agitation, anhedonia (refusal to play, isolation), feelings of worthlessness (“no one likes me,” “I’m no good”), secondary diurnal enuresis, suicidal thoughts (threats of defenestration), and need for sertraline (50 mg/day) treatment and psychotherapy. There was complete remission of the symptoms after six months.

In the last two years, she began to present episodes of emotional dysregulation, with altered states of consciousness but always in an awake state, sialorrhea, loss of urinary sphincter control, auto- and hetero-aggressions, and uncontrollable crying for one to two hours, always when someone was nearby, and never with tongue biting. Afterward, she would return to a steady state, frequently with a need to sleep or rest. A vigil and sleep video electroencephalography was requested after the first episode, and it showed no paroxysmal activity.

The frequency of the episodes increased to up to three per week, with a great impact on her daily life and school performance. Risperidone (1 mg/day) was introduced when she was 12 years old, with a moderate response (one episode per week), and later, aripiprazole (15 mg/day) was associated with a good response (one episode per month).

Recently, the episodes of emotional dysregulation and exuberant behavioral problems returned more frequently, without apparent precipitating events. The disruption in her routines caused by these moments, including school absence, required psychopharmacological intervention with a switch to paliperidone (6 mg/day) and initiation of valproate semisodium (1000 mg/day) in association with behavioral techniques.

Lately, during these events, the girl presented with disorganized speech, derailment, incoherence, perseveration of speech, grossly disorganized behavior, with auto- and hetero-aggression. Sometimes, auditory hallucinations were apparent (‘he said my mom died,’ ‘he told me to cut myself’) but difficult to evaluate, with good response to intramuscular or oral chlorpromazine (25 mg) and a return to a steady state afterward.

Major school adaptations and weekly group therapy were provided, with a good response. Nowadays, she has less than an episode per week and is attending school while still on the latest drugs introduced (paliperidone and valproate semisodium). Also, the caretakers are now able to contain these behaviors without additional pharmacological aid.

## Discussion

The 3q29 microduplication syndrome was described for the first time in a small family that presented with intellectual disability and dysmorphic alterations [2]. Since then, 35 cases were reported in the literature [1-11]. However, the psychiatric symptoms in these patients were not extensively described. Most of these individuals present cognitive disabilities and developmental delays. One was diagnosed with autism spectrum disorder and another one with attention-deficit/hyperactivity disorder [1,7]. Those two also presented with behavioral disorders [1,7]. There is also information on elimination disorders in two cases [1,4].

This case reports an unusual presentation of the 3q29 microduplication syndrome.

In our opinion, the differential psychiatric diagnosis for this late complex symptomatology, according to the Diagnostic and Statistical Manual of Mental Disorders - 5th edition (DSM-5), is brief psychotic disorder, bipolar II disorder, posttraumatic stress disorder, and disruptive mood dysregulation disorder [13].

To assume a brief psychotic disorder, we can confirm at least two of the four possible symptoms (disorganized speech and grossly disorganized behavior) and the return to the premorbid level of functioning. Other associated features that were present and that support this diagnosis is the emotional turmoil, knowing that this can be the first presentation of a psychotic disorder in adolescence and usually in females and that this girl presented with high levels of suspicion of persecution. However, the duration of the episodes is less than one day, which excludes this diagnosis.

The possibility of an early-onset bipolar II disorder exists because this girl had presented with a major depressive episode (described above) in the past and had a distinct period of irritable mood. However, this mood was not comorbid with abnormal and increased activity or energy for at least four consecutive days, and there were no other symptoms of a hypomanic episode, which excludes the diagnosis. Nevertheless, since this disorder is usually diagnosed in later adolescence and throughout adulthood, we cannot assume that this diagnosis is not a possibility in the future.

The inclusion of posttraumatic stress disorder in the differential diagnosis is based on the repeated trauma experienced by this girl while under her parents’ care. These episodes of dysregulation could be interpreted as flashbacks, and the irritable behavior and angry outbursts could also be explained by this condition. However, neither of the other necessary criteria is met for this diagnosis.

The disruptive mood dysregulation disorder diagnosis implies severe recurrent temper outbursts that can be manifested behaviorally and that are grossly out of proportion to the intensity or duration of the situation, that is inconsistent with the developmental level, occurs on average more than three times a week, and the mood between the outbursts needs to be persistently irritable most of the day, nearly every day, and observable by others. Additionally, the outbursts and the irritable mood need to be present in at least two settings and be severe in one. However, the recent symptoms of disorganized speech with derailment, incoherence, and perseveration of speech, and grossly disorganized behavior, with auto- and hetero-aggression and apparent hallucinations, cannot be explained by this.

The DSM-5 proposed criteria for attenuated psychosis syndrome (attenuated hallucinations or disorganized speech at least once a week in the past month, worsened in the last year, and needing clinical attention) could be considered in this girl, but it should not be used for clinical purposes.

In the case that we presented, both pharmacological and non-pharmacological interventions were needed. Since the start, emotional support and regular psychiatric consultations were provided, helping the caretakers to understand and deal with these episodes. Behavioral techniques were taught to the caretakers and schoolteachers to limit these moments in time. For instance, it was important to isolate the girl from her peers during these episodes and to take her into a place where she could not hurt herself or others, offering her adult support and containing her psychomotor agitation. Social adaptations were also made, first with supervised parental visitations to the girl and now with no visitations at all (parental and court decisions).

Pharmacological therapy was initiated with the consent of the girl’s legal guardian. Atypical antipsychotics were used as the first line, given the efficacy in managing psychomotor agitation. The mood stabilizer was then used as an adjuvant, given the level of emotional dysregulation and its efficacy in behavioral alterations.

Recently, the school was able to include the girl in a smaller class with a special educational needs teacher and to reduce the number of hours in her schedule since it was clear that she was getting frustrated with the high demands. One of her caretakers also began to go to the school around lunchtime, to give some support and to help motivate the girl for the rest of the day.

The child and adolescent psychiatry department included her in the day hospital unit, with weekly group therapy, improving her social skills, and providing emotional and technical support. This multimodal method is helping this girl reduce the outbursts and alleviate the pharmacologically acute treatment. No semi-structured or structured interviews or interventions were applied, and this is a limitation to our approach.

## Conclusions

In conclusion, even though this girl was diagnosed with a moderate intellectual developmental disorder, which is already reported in other cases, she had unique features in her psychiatric symptoms. To our knowledge, she is the only one who was also diagnosed with a depressive disorder. Besides this, she was the only case with a tic disorder. Nevertheless, the unique features appear to be related to her emotional dysregulation, which required major adaptations in her daily life with pharmacological and non-pharmacological interventions, and, in our opinion, cannot be defined by any diagnostic classification.
